# Zebrafish *etv7* regulates red blood cell development through the cholesterol synthesis pathway

**DOI:** 10.1242/dmm.012526

**Published:** 2013-12-19

**Authors:** Anita M. Quintana, Fabrizio Picchione, Ramon I. Klein Geltink, Michael R. Taylor, Gerard C. Grosveld

**Affiliations:** 1 St Jude Children’s Research Hospital, Department of Genetics, 262 Danny Thomas Place, Memphis, TN 38105, USA.; 2 St Jude Children’s Research Hospital, Department of Chemical Biology & Therapeutics, 262 Danny Thomas Place, Memphis, TN 38105, USA..

**Keywords:** Cholesterol, *etv7*, Oncogene, Red blood cell

## Abstract

ETV7 is a human oncoprotein that cooperates with Eμ-MYC to promote pre-B-cell leukemia in mice. It is normally expressed in the bone marrow and fetal liver and is upregulated in primary leukemia, suggesting that it is involved in proper hematopoiesis and leukemogenesis. *ETV7* has been deleted in most rodents, but is conserved in all other vertebrates, including the zebrafish, *Danio rerio*. In this report, we characterize the function of the zebrafish *etv7* gene during erythropoiesis. Our results demonstrate that *etv7* regulates the expression of the zebrafish *lanosterol synthase* (*lss*) gene, an essential gene in the cholesterol synthesis pathway. Furthermore, morpholino knockdown of *etv7* leads to loss of hemoglobin-containing red blood cells, a phenotype that can be rescued by injection of exogenous cholesterol. We conclude that *etv7* is essential for normal red blood cell development through regulation of the *lss* gene and the cholesterol synthesis pathway.

## INTRODUCTION

*ETV7* is a human oncogene that causes leukemia when expressed in murine bone marrow (Cardone et al., 2005; Carella et al., 2006). It is an E26 transformation specific (ETS) factor that is mostly expressed in the human hematopoietic system. *ETV7* was originally identified by three independent groups and is highly homologous to *ETV6* (Gu et al., 2001; Poirel et al., 2000; Potter et al., 2000). The ETV6 and ETV7 proteins each belong to the TEL/Yan subclass of ETS transcription factors and have a highly conserved ETS DNA-binding domain (ETS domain) and a pointed (PNT) protein-protein interaction domain (Slupsky et al., 1998).

In humans, *ETV7* is primarily expressed in the bone marrow and fetal liver, and has been implicated in the regulation of hematopoiesis. Our laboratory demonstrated that, in the U937 human monocytic cell line, expression of *ETV7* decreases upon vitamin-D3-induced differentiation (Kawagoe et al., 2004), suggesting that the expression level of *ETV7* is highly regulated during the differentiation process. Moreover, forced expression of *ETV7* in murine bone marrow causes a latent myeloproliferative disease that is dependent on the cooperation of secondary mutations (Carella et al., 2006). One example of a secondary mutation capable of cooperating with *ETV7* during transformation is the *Eμ-MYC* allele: it has been established that overexpression of *ETV7* in murine bone marrow harboring this allele accelerates pre-B-cell lymphomagenesis (Cardone et al., 2005). Taken together, these data suggest that *ETV7* might play an important role during normal hematopoiesis and leukemia.

Although previous work has provided valuable information about the potential oncogenic role of *ETV7*, they have not addressed the physiological role of endogenous *ETV7*. In order to determine the physiological role of *ETV7*, we developed a novel *in vivo* developmental model using the zebrafish, *Danio rerio*. This model is unique because most rodents, including mice, have deleted the endogenous *ETV7* gene. Using zebrafish to study *etv7* function, we show that loss of *etv7* leads to a marked reduction in hemoglobinized red blood cells, which is mediated indirectly through the cholesterol synthesis pathway. Here we provide evidence for the efficacy of this new model and for the newly identified role of *etv7* in the cholesterol biosynthesis pathway.

## RESULTS

### The human and zebrafish *etv7* genes have overlapping expression patterns

**Fig. 1.**
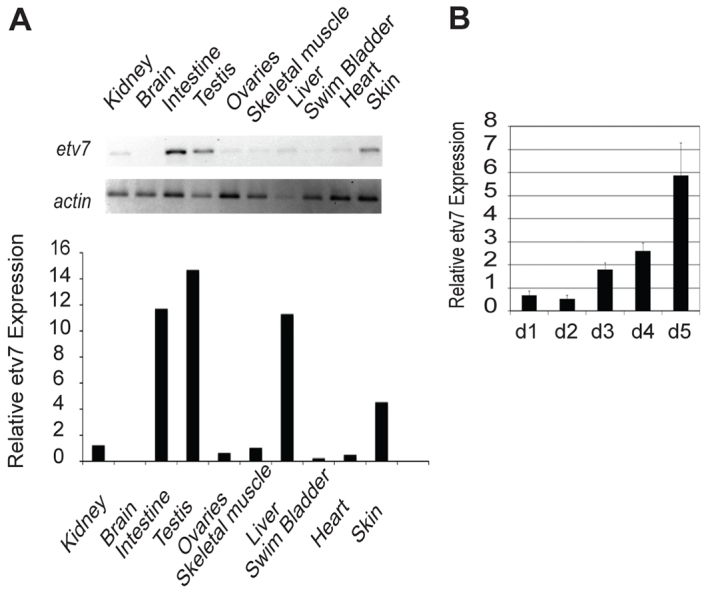
***etv7* is expressed in the adult and developing zebrafish.** (A) Semi-quantitative RT-PCR of *etv7* mRNA of adult zebrafish tissues. *actin* is provided as a positive loading control. Quantification of the *etv7* signals relative to *actin* is shown below. (B) Quantitative real-time PCR measuring the level of *etv7* expression during the first 5 days of development. All values are relative to day 0, which is 4 hpf. d1=day 1 post-fertilization etc. Error bars represent standard deviation.

The goals of this work were: (1) to employ an appropriate animal model to study *etv7* function, and (2) to determine the function of *etv7* during development. Zebrafish provide a unique means of determining *etv7* gene function because the gene is highly conserved and loss-of-function studies cannot be done in the mouse because it does not have the gene. Because human *ETV7* is expressed in a variety of adult tissues (Gu et al., 2001), we performed semi-quantitative PCR on adult zebrafish tissues and demonstrated relatively high expression in the intestine, testes and liver, whereas all other organs examined had a much lower level of expression (Fig. 1A). Of the tissues examined, only the brain did not express *etv7*. The low level of expression in most tissues is consistent with other studies (Gu et al., 2001). Previous work also demonstrated that *ETV7* is expressed developmentally (Gu et al., 2001; Potter et al., 2000). Quantitative real-time PCR analysis demonstrated that zebrafish *etv7* expression increased ~5.8-fold by 5 days post-fertilization (dpf) (Fig. 1B). In addition, *in situ* hybridization of animals at 1, 2, 3 and 4 dpf confirmed that *etv7* was developmentally expressed (supplementary material Fig. S1). These data demonstrate that *etv7* is expressed during development and into adulthood.

### Loss of *etv7* causes a reduction in hemoglobinized red blood cells

**Fig. 2.**
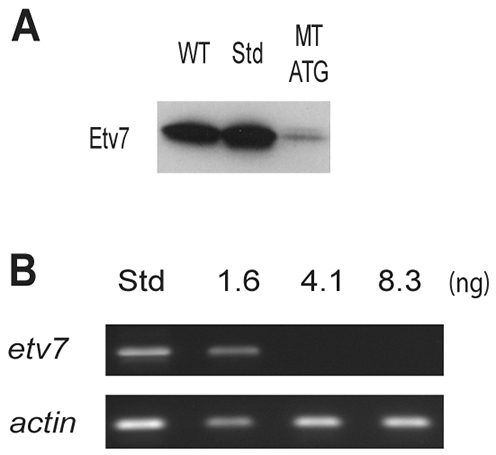
**Morpholino-mediated knockdown of *etv7***. (A) *In vitro* transcription/translation assay analyzing the specificity of a translation-inhibiting morpholino. Western blot was performed with anti-HA antibodies using an HA-tagged version of *etv7*. WT is the normal reaction without any morpholino present, Std is the assay with 8.2 ng of standard control morpholino and MT ATG is the assay with 8.2 ng of translational-inhibiting morpholino. (B) Semi-quantitative PCR demonstrating the efficacy of the splice-site-inhibiting morpholino. Concentrations of morpholinos are in nanograms and Std represents injection with 8.2 ng of standard control morpholino. *actin* is included as a non-specific loading control.

The low level of *etv7* expression in multiple tissues might indicate a fundamental role of *etv7* during both development and adulthood. To address the role of *etv7* during development, we performed morpholino-oligonucleotide-mediated knockdown and examined the developing embryos. Two independent morpholinos were designed: one that inhibited translation and one that inhibited proper splicing of exon 3, which encodes most of the PNT domain. The efficacy of the translation-blocking morpholino (MT ATG) was assessed with *in-vitro*-translated Etv7-tagged with hemagglutinin (HA). The efficacy of the pre-mRNA splicing morpholino (MT Splice) was assessed by RT-PCR. Adding the MT ATG morpholino to an *in vitro* transcription/translation system with *etv7* cDNA demonstrated a marked reduction in Etv7 protein synthesis as measured by western blot analysis, whereas adding a standard control morpholino to this system had no effect on the production of Etv7 protein (Fig. 2A). In addition, injection of the MT Splice morpholino caused a dose-dependent reduction in the level of *etv7* mRNA, but had no effect on the level of control *actin* mRNA (Fig. 2B). Together, these data established that each morpholino specifically reduces the level of endogenous *etv7*.

**TRANSLATIONAL IMPACT**

**Clinical issue**

The human E26 transformation-specific (ETS) transcription factor *ETV7* is normally expressed mainly in bone marrow and fetal liver, and is highly regulated during differentiation. Notably, *ETV7* is upregulated in a variety of different human cancers and, when introduced into murine bone marrow, can cooperate with other oncogenes to cause hematopoietic malignancy. Thus, it seems that *ETV7* is involved in hematopoiesis and in leukemogenesis. However, although significant progress has been made in establishing *ETV7* as an oncogene, little is known about the normal function of this gene in development and adulthood, in part because of the lack of an appropriate model system – *Etv7* has been deleted in most rodent lineages, including the mouse.

**Results**

ETV7 is highly conserved in zebrafish, which is an established model for the study of hematopoiesis. In this study, therefore, the authors use zebrafish to study the physiological role of ETV7. The authors show that *etv7* is expressed during development and throughout adulthood in zebrafish, which is consistent with previous reports of ETV7 expression in humans. Microarray analysis of embryos transiently overexpressing Etv7 indicates that Etv7 regulates the expression of the zebrafish *lanosterol synthase* (*lss*) gene, an essential gene in the cholesterol synthesis pathway. Finally, the authors use a morpholino-based approach to demonstrate that loss of *etv7* in zebrafish results in a severe reduction in the number of hemoglobin-containing red blood cells, a phenotype that can be rescued by injection of exogenous cholesterol.

**Implications and future directions**

These findings demonstrate that *etv7* is essential for normal red blood cell development in zebrafish and suggest that it acts through regulation of *lss* and the cholesterol synthesis pathway. Importantly, these findings identify zebrafish as an appropriate animal model in which to study *etv7* function. Specifically, although previous work characterizing this important oncogene in cell culture and mice has provided valuable information, the zebrafish could provide a more relevant *in vivo* developmental model for this gene in which key questions regarding the molecular and cellular mechanisms regulated by *etv7* can be addressed. Uncovering these mechanisms could lead to the development of new models in which to characterize the role of *etv7* during development and disease, and to the discovery of new cancer therapies.

**Fig. 3.**
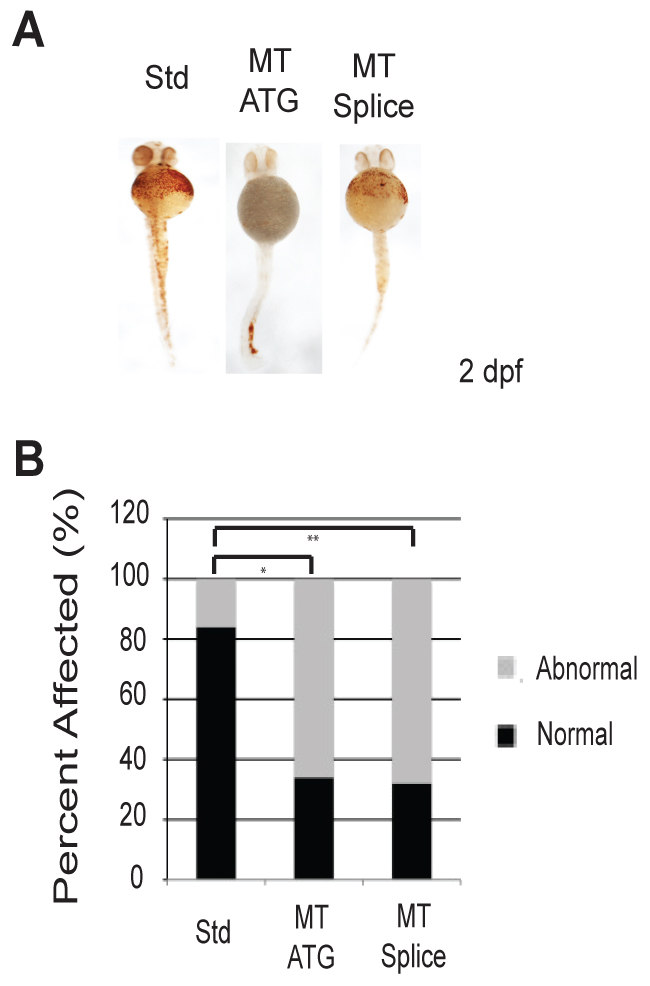
**Loss of *etv7* causes a marked reduction in hemoglobinized red blood cells.** (A) *etv7* morphants stained with *o*-dianisidine at 2 dpf. Std represents embryos injected with 8.2 ng of standard control morpholino, MT ATG are embryos injected with 8.2 ng of translation-blocking morpholino, and MT Splice are embryos injected with 4.1 ng of splice-site morpholino. Normal embryos were classified by the presence of adequate heme staining according to *o*-dianisidine, and fish with reduced heme staining are classified as abnormal. (B) Quantitation of A (MT ATG *n*=44, Std *n*=44, MT Splice *n*=34). *P≤0.0001, **P≤0.0001.

ETV7 has been shown to regulate hematopoiesis and in humans it is expressed in the fetal liver and bone marrow (Cardone et al., 2005; Carella et al., 2006; Kawagoe et al., 2004; Poirel et al., 2000; Potter et al., 2000). Analysis of adult zebrafish tissue confirmed that *etv7* is expressed in the zebrafish kidney, a site of hematopoiesis equivalent to mammalian bone marrow (Zapata, 1979). The expression of *etv7* in the zebrafish kidney raised the possibility that loss of *etv7* might cause defects in hematopoiesis. Etv7 morphants exhibited reduced staining of hemoglobinized red blood cells at 2 dpf, which is consistent with the idea that loss of *etv7* causes defects in red blood cell ontogeny (Fig. 3A). Loss of heme staining was observed in morphants injected with either the MT ATG or the MT Splice morpholino. Both morpholinos caused a loss of hemoglobinized red blood cells in ~65–75% of animals injected (Fig. 3B). Morpholino injection with both the MT ATG and the MT Splice resulted in different degrees of phenotype penetrance. An example of the degrees of severity for the MT ATG morpholino included: (1) embryos with no detectable hemoglobinized red blood cells (23.7%, *n*=14), (2) embryos with a reduced number of red blood cells (47.4%, *n*=28), and (3) embryos with normal numbers of red blood cells (28.9%, *n*=17). In addition, injection of each morpholino into p53-deficient embryos did not change the degree or penetrance of the phenotype, providing evidence that the phenotype was p53-independent (supplementary material Fig. S2A,B) (Gerety and Wilkinson, 2011). These data were independently confirmed with an injection of an additional morpholino inhibiting pre-mRNA splicing at exon 5 (supplementary material Fig. S3A,B) (*n*=50, 43% penetrance). A standard control morpholino did not cause a red blood cell phenotype at any of the tested concentrations. Together, these data indicated that this phenotype is a direct result of the loss of *etv7* and not a result of non-specific off-target effects.

### *etv7* morphants maintain *beta-globin* mRNA expression during development

**Fig. 4.**
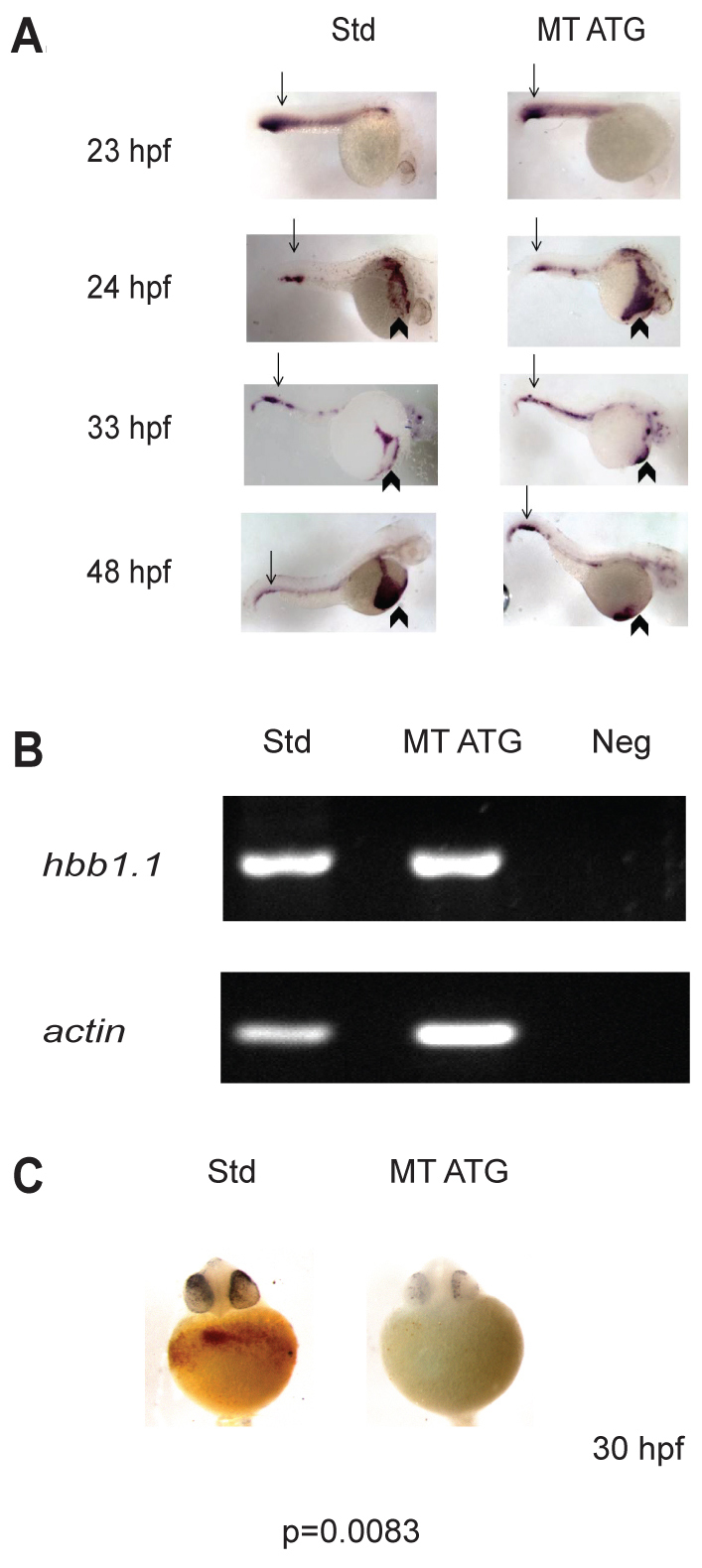
***etv7* morphants express *beta-globin* (*hbbe1.1*).** (A) *In situ* hybridization analyzing the expression of *beta-globin* mRNA was performed on embryos injected with 8.2 ng of standard control morpholino (Std) or 8.2 ng of translational-blocking morpholino (MT ATG) at 23, 24, 33 or 48 hpf. Arrows indicate expression in the posterior blood island (PBI) and arrowheads depict expression on the yolk sac. (B) Semi-quantitative PCR of *beta-globin* (*hbbe1.1*) expression at 24 hpf. *actin* expression is shown as loading control. Std are animals injected with 8.2 ng of standard control morpholino, MT ATG are animals injected with 8.2 ng of translational blocking morpholino and Neg is control PCR reaction without template. (C) *etv7* morphants exhibit reduced levels of mature red blood cells. *o*-dianisidine staining was performed at 30 hpf with embryos injected with 8.2 ng of translational-blocking morpholino (MT ATG) or 8.2 ng standard control morpholinos (Std).

The absence of hemoglobinized red blood cells could result from the inability to express *beta-globin*. To determine whether the loss of hemoglobinized red blood cells results from defects in *beta-globin* mRNA expression, *in situ* hybridization at 23, 24, 33 and 48 hours post-fertilization (hpf) was performed on embryos injected with the standard control morpholino or MT ATG. We found that *beta-globin* (*hbbe1.1*) mRNA was equivalently expressed in control and morphant animals at each time point examined (Fig. 4A). These data suggest that a loss of hemoglobinized red blood cells is not due to the inability to express *beta-globin* mRNA. Furthermore, our analysis demonstrated that *beta-globin*-positive cells are localized to the correct regions before and after the onset of circulation, ruling out the possibility that *etv7* knockdown results in circulation defects (Fig. 4A, 23 and 24 hpf). Semi-quantitative PCR at 24 hpf confirmed that *beta-globin* expression did not change in morphant animals (Fig. 4B). Furthermore, a reduction in hemoglobinized red blood cells was evident as early as 30 hpf in morphant animals (*n*=29) relative to standard control-injected animals (*n*=23) (Fig. 4C). Approximately, 45% of the animals injected with MT ATG exhibited blood cell defects at 30 hpf (*P*=0.0083). These data are consistent with the hypothesis that knockdown of *etv7* interferes with red blood cell differentiation independent of *beta-globin* expression.

### *gata1* mRNA expression is normal in morphants

**Fig. 5.**
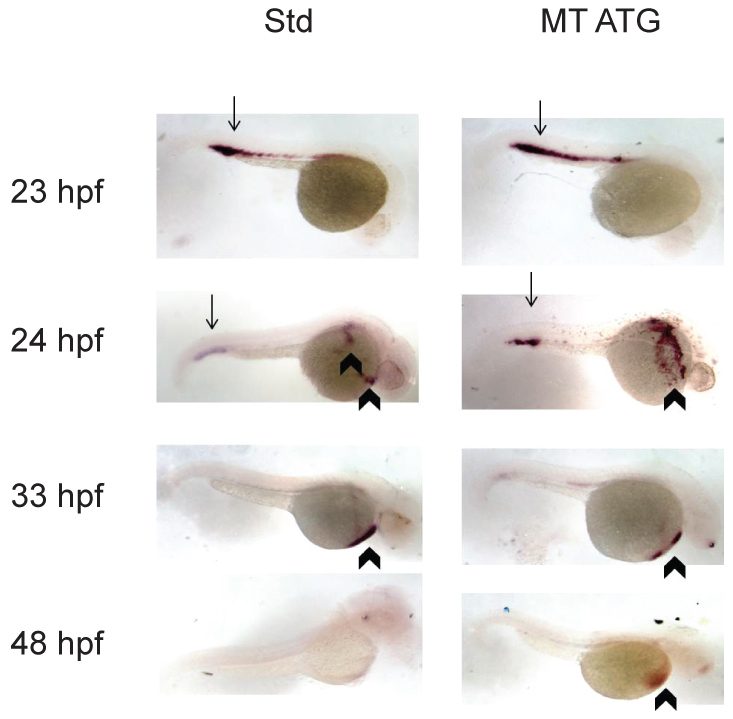
***gata1* expression is maintained at 48 hpf in morphants.** *In situ* hybridization detecting *gata1* mRNA expression at 23, 24, 33 and 48 hpf in standard control-injected embryos (Std) or *etv7* translational-blocking morpholinos (MT ATG). 8.2 ng of each morpholino was injected. Arrows indicate expression in the posterior blood island (PBI) and arrowheads depict expression on the yolk sac.

Defects in proper specification of red blood cells could result in an absence of mature red blood cells. To begin to address this possibility, *in situ* hybridization detecting *gata1* mRNA expression was performed at 23, 24, 33 and 48 hpf. Prior to the onset of circulation, *gata1* expression was located in the PBI (peripheral blood island) and ICM (intermediate cell mass) of both morphants and control animals. At the onset of circulation, ~24 hpf, *gata1* expression was observed in the PBI and on the yolk sac with no significant difference between morphant and control animals. At 33 hpf, after the onset of definitive hematopoiesis, *gata1* expression was observed on the yolk sac of both control and morphant animals. However, at 48 hpf, *gata1* expression was retained on the yolk sac of morphants (*n*=40), whereas there was no detectable *gata1* expression in the embryos injected with standard control morpholinos (*n*=29) (Fig. 5). Approximately 72% of the embryos analyzed demonstrated this abnormal pattern of *gata1* expression relative to control (*P*<0.0001).

### Etv7 regulates red blood cell development through the cholesterol synthesis pathway

**Fig. 6.**
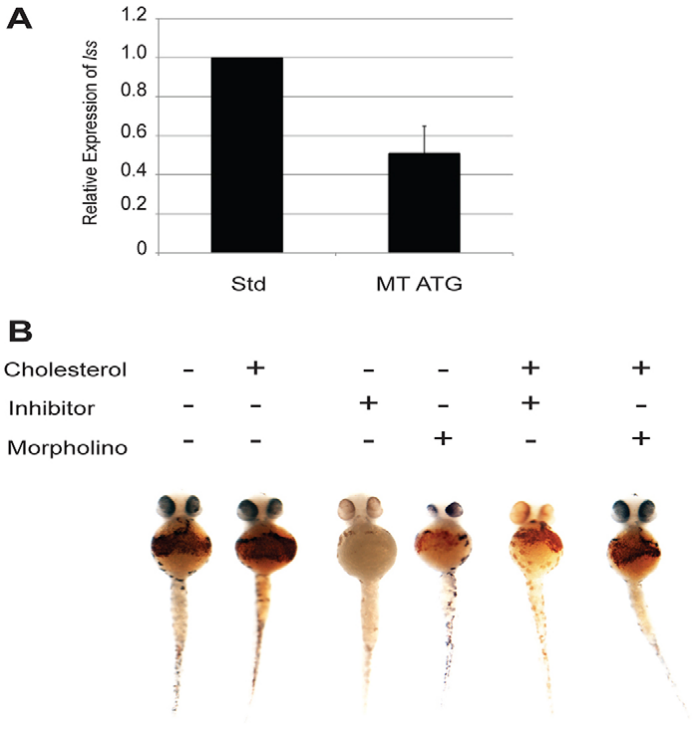
***etv7* regulates red blood cells through the cholesterol synthesis pathway.** (A) Real-time PCR analysis of *lss* expression in embryos injected with either 8.2 ng of standard control morpholino (Std) or 8.2 ng of *etv7* morpholinos (MT ATG). Error bars represent standard deviation. (B) Inhibition of Lss enzymatic activity phenocopies knockdown of *etv7*. Wild-type embryos were treated with the Lss inhibitor Ro 48-8.071 (50 nM) and stained with *o*-dianisidine at 2 dpf. Injection of *etv7* morpholinos was performed simultaneously and compared with embryos treated with Ro 48-8.071. Rescue experiments were performed by injecting cholesterol into the yolk of embryos at 1 dpf. *o*-dianisidine staining was used to visualize the presence or absence of hemoglobinized red blood cells at 2 dpf (Std *n*=26, Std with cholesterol *n*=32, MT ATG *n*=20, MT ATG with cholesterol *n*=15). Refer to Table 1 for *P*-values and percent affected in each category.

Etv7 is a transcription factor and therefore it is plausible that Etv7 regulates red blood cell development indirectly via downstream target genes. To test this idea, microarray analysis on embryos transiently overexpressing Etv7 was performed in order to identify possible downstream target genes. This analysis revealed that Etv7 regulates expression of the zebrafish *lanosterol synthase* (*lss*) gene (supplementary material Table S1). Morpholino-mediated loss of *etv7* resulted in a significant decrease of *lss* mRNA (Fig. 6A). Furthermore, drug inhibition of Lss enzyme activity with Ro 48-8.071, an Lss-specific inhibitor, resulted in a loss of hemoglobinized red blood cells (Fig. 6B; Table 1). LSS regulates the final step in the cholesterol synthesis pathway and has been shown to regulate the self-renewal of chicken erythrocyte progenitors (Mejia-Pous et al., 2011). If Etv7 regulates red blood cell development through the cholesterol synthesis pathway, then we predicted that administration of exogenous cholesterol to *etv7* morphants should rescue the observed phenotype. Indeed, injection of exogenous cholesterol restored wild-type levels of hemoglobinized red blood cells in the *etv7* morphants (Fig. 6B). This result indicated that exogenous cholesterol compensates for loss of endogenous cholesterol synthesis due to the reduction in *lss* expression and adequately rescues the phenotype associated with loss of *etv7*. Furthermore, we found that *lss* was expressed in a similar subset of tissues as was *etv7* (supplementary material Fig. S4). Taken together, these data provide evidence that loss of *etv7* leads to reduced expression of *lss*, which in turn affects red blood cell development.

**Table 1.**
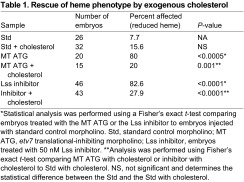
Rescue of heme phenotype by exogenous cholesterol

* Statistical analysis was performed using a Fisher’s exact *t*-test comparing embryos treated with the MT ATG or the Lss inhibitor to embryos injected with standard control morpholino. Std, standard control morpholino; MT ATG, *etv7* translational-inhibiting morpholino; Lss inhibitor, embryos treated with 50 nM Lss inhibitor.

** Analysis was performed using Fisher’s exact *t*-test comparing MT ATG with cholesterol or inhibitor with cholesterol to Std with cholesterol. NS, not significant and determines the statistical difference between the Std and the Std with cholesterol.

## DISCUSSION

*ETV7* has been shown to promote tumorigenesis in mice and previous work has characterized the effects of *ETV7* overexpression in murine bone marrow. However, a comprehensive understanding of the mechanisms via which *ETV7* mediates tumorigenesis is lacking and has been impeded by the lack of an appropriate animal model. Part of the rodent lineage has deleted the *Etv7* gene; however, all other vertebrates, including zebrafish, have retained the gene. Here we used zebrafish as an appropriate animal model to study *ETV7* function and provide evidence that *etv7* regulates red blood cell development indirectly through the cholesterol synthesis pathway.

Analysis of the expression patterns in zebrafish demonstrated that *etv7* is expressed both developmentally and throughout adulthood. The expression level of *etv7* was low across most adult tissues, an observation that is consistent with previously published work using human tissues (Gu et al., 2001). To examine the role of *etv7* during development, we used a morpholino-based approach. The most obvious phenotype of *etv7* knockdown was a marked reduction in the number of hemoglobinized red blood cells. A hematopoietic defect is not an unanticipated result because human *ETV7* is expressed in the bone marrow and fetal liver (Potter et al., 2000). Here we have focused entirely on red blood cell development, given that preliminary analysis of markers associated with other lineages such as *PU.1* and *scl* did not indicate additional blood cell defects. In the future, a more robust analysis of other cell types will need to be performed to potentially uncover a wider role of *etv7* during zebrafish hematopoiesis.

Although little is known about the endogenous role of *ETV7*, an animal model for the highly related *ETV6* gene has been characterized. Loss of *Etv6* in the mouse causes defects in hematopoiesis (Wang et al., 1998), whereas overexpression of the *Etv6* gene in erythroid cells leads to enhanced proliferation and increased hemoglobin synthesis (Eguchi-Ishimae et al., 2009). Given the phenotypes associated with the gain or loss of function of both *ETV6* and *ETV7*, which can physically interact (Potter et al., 2000), it is plausible that these two proteins have some overlapping functions. However, *ETV6* and *ETV7* do not completely overlap in functionality because *ETV6* inhibits cell proliferation and transformation (Kawagoe et al., 2004), whereas *ETV7* is a known oncogene (Carella et al., 2006).

This study provides evidence that *etv7* directly or indirectly regulates the expression of *lss*, a gene involved in cholesterol biosynthesis. Recent work has demonstrated that LSS is essential for the self-renewal of chicken erythroid precursor cells (Mejia-Pous et al., 2011). In this study, the authors demonstrated that LSS was important for maintaining the self-renewal capacity of red blood cells, but LSS inhibition by Ro 48-8.071 did not affect the function of fully differentiated cells. Furthermore, maintenance of self-renewal was directly dependent upon the presence of cholesterol. LSS is highly conserved between chicken and zebrafish. The enzymatically active squalene cyclase domain (measuring 639 amino acids) is 77% identical and 85% homologous between the two species. Thus, there is little doubt that the Ro 48-8.071-induced loss of hemoglobinized red blood cells in zebrafish is due to inhibition of Lss, a result that was phenocopied by *etv7* knockdown. We further demonstrated that the loss of hemoglobinized red blood cells in *etv7* morphants was dependent upon the presence of cholesterol, given that administration of exogenous cholesterol rescued the phenotype. The red blood cell membrane is composed of both cholesterol and phospholipids; thus, cholesterol is likely to be an essential mediator of red blood cell development (Mohandas and Gallagher, 2008). Our combined analysis of *beta-globin* expression, *gata1* expression and *o*-dianisidine staining suggests that *etv7* is important for red blood cell development. In the future, it will be necessary to gain a more complete understanding of the role that cholesterol has during the differentiation and specification of red blood cells. These data, together with reduced *lss* expression upon *etv7* knockdown, substantiate the previously known role of *lss* and cholesterol in erythrocytes and highlights the unappreciated role of *etv7* in the cholesterol biosynthesis pathway.

In this study, we demonstrated that zebrafish provide a unique model to study *etv7* gene function. We addressed the function of *etv7* during development, but we have not addressed the role of *etv7* during tumorigenesis. It will be interesting to determine whether *etv7* regulates cholesterol biosynthesis in other cell types and during tumorigenesis. Zebrafish have been used in the past to study the role of specific oncogenes in cancer development and it has been shown that zebrafish develop histologically similar cancer phenotypes as mice and humans. For example, *AML1:ETO*, *TEL1:JAK2A* and *TEL1:AML1* transgenic zebrafish have been generated, which closely phenocopy the results obtained in mouse models (reviewed in Quintana and Grosveld, 2011). There is now a need to gain a more complete understanding for the effects of *etv7* overexpression and the potential role of this gene in a zebrafish cancer model system.

## MATERIALS AND METHODS

### Zebrafish and maintenance

Zebrafish (*Danio rerio*) were maintained at St Jude Children’s Research Hospital according to the Institutional Animal Care and Use Committee (IACUC) guidelines. For all experiments, zebrafish embryos [Tupfel long fin (TL) or tp53^zdf1^] were maintained in egg water consisting of 0.03% Instant Ocean (Aquarium Systems, Inc., Mentor, OH) in R.O. water at 28.5°C.

### RNA isolation and PCR analysis

For RNA isolation, larvae were manually dechorionated, lysed in Trizol (Invitrogen, Grand Island, NY) (200 μl), and processed according to the manufacturer’s recommendations. RNA (3 μg) was converted into cDNA with Superscript III Reverse Strand Synthesis system (Invitrogen, Grand Island, NY) according to manufacturer’s protocol with random hexamer primers. Semi-quantitative PCR was performed with GoTaq mastermix (Promega, Madison, WI). Sequences of primers are listed in supplementary material Table S2.

### *In vitro* knockdown analysis and western blot

For *in vitro* transcription/translation, 360 ng pGEM-*etv7* DNA was used to program the TNT SP6 Quick Coupled Transcription/Translation system (Promega, Madison, WI) according to the manufacturer’s instructions. Western blots were probed with anti-HA antibody according to manufacturer’s protocol (Cell Signaling Technology, Danvers, MA). Western blots were developed with SuperSignal West Pico Chemiluminescent substrate (Pierce, Rockford, IL).

### Morpholino injection and analysis

Tupfel long fin (TL) or *tp53^zdf1^* larvae were injected with 8.2 ng of standard control morpholino (5′-CCTCTTACCTCAGTTACAATTTATA-3′), 8.2 ng of translational blocking morpholino (5′-GTGAAGAGGCGTCACTCATGTTCTT-3′), 4.1 ng of *etv7* splice site exon 3 morpholino (5′-GATGCCTGCACATTATTTTCATCTT-3′) or 8.2 ng of *etv7* splice exon 5 (5′-GACCTGCAAAACCAATTATTGCTGT-3′) (Gene Tools, LLC, Philomath, OR). A range of concentrations from 2.0 to 16.4 ng of standard control morpholino was injected to determine that the standard control morpholino did not cause any blood cell defects. For subsequent experiments an equal concentration of standard control and *etv7*-specific morpholinos was used and is specified in the manuscript figure legends. *o*-dianisidine (Sigma, St Louis, MO) staining was performed as previously described (Paffett-Lugassy and Zon, 2004). Statistical analysis was performed according to a Fisher’s exact *t*-test with the online software calculator from Graphpad Prism.

### Etv7 overexpression and DNA microarray analysis

DNA (200 pg/embryo) was injected into single-cell embryos. At 1 dpf, embryos (10) were harvested and total RNA was isolated as described under the ‘RNA isolation and PCR analysis’ heading. Total RNA was hybridized to the Zebrafish Gene Expression Microarray (Agilent Technologies, Santa Clara, CA). Each experiment was performed with biological duplicates. All microarray experiments and analyses were performed by the Hartwell Center for Bioinformatics and Biotechnology core facility at St Jude Children’s Research Hospital.

### Tissue isolation and RNA analysis

Adult zebrafish were sacrificed in 0.04% tricaine solution and various organs and tissues were dissected out. Organs were lysed in Trizol (Invitrogen, Grand Island, NY) (200 μl) and processed according to the manufacturer’s recommendations. RNA was converted into cDNA with Superscript III Reverse Strand Synthesis system (Invitrogen, Grand Island, NY) with oligo dT primers according to manufacturer’s protocol. Semi-quantitative PCR was performed with GoTaq mastermix (Promega, Madison, WI) and sequences of primers are listed in supplementary material Table S2.

### *In situ* hybridization

All *in situ* hybridization was performed as previously described (Thisse and Thisse, 2008). Briefly, embryos were harvested at the indicated time point and fixed in 4% paraformaldehyde (Sigma, St Louis, MO) overnight at 4°C. Each sample was washed in PBS with 0.1% Tween 20 (Sigma, St Louis, MO) (PBT) and permeabilized with proteinase K for varying amounts of time according to developmental stage. All probes were hybridized overnight, except the *etv7* probe, which was hybridized for 72 hours, and washed in wash solution containing 50% formamide (Sigma, St Louis, MO). Samples were blocked in 20% sheep serum (Sigma, St Louis, MO) for 4–5 hours and incubated with anti-DIG Fab fragments (Roche, Indianapolis, IN) overnight. All samples were developed with the BM Purple AP substrate (Roche, Indianapolis, IN) and imaged by conventional microscopy.

### Lss inhibition and cholesterol injection

Lss was inhibited with 50 nM Ro 48-8.071 by addition directly to egg water at 4 hpf, followed by incubation until 2 dpf. Cholesterol (Sigma, St Louis, MO) was injected at 2 μg/μl into the yolk sac of embryos at 1 dpf. For rescue experiments, embryos were injected with cholesterol after 24 hours incubation with 50 nM Ro 48-8.071 (Sigma, St Louis, MO). After injection, embryos were incubated an additional 24 hours in 50 nM Ro 48-8.071. To rescue the *etv7*-specific phenotype, embryos were injected with morpholinos at the single-cell stage and then injected with cholesterol at 1 dpf. All embryos were analyzed at 2 dpf with *o*-dianisidine according to the protocol described by Paffett-Lugassy and Zon (Paffett-Lugassy and Zon, 2004).

## References

[b1-0070265] Cardone M. Kandilci A. Carella C. Nilsson J. A. Brennan J. A. Sirma S. Ozbek U. Boyd K. Cleveland J. L. Grosveld G. C. (2005). The novel ETS factor TEL2 cooperates with Myc in B lymphomagenesis. Mol. Cell. Biol. 25, 2395–2405 15743832 10.1128/MCB.25.6.2395-2405.2005 PMC1061619

[b2-0070265] Carella C. Potter M. Bonten J. Rehg J. E. Neale G. Grosveld G. C. (2006). The ETS factor TEL2 is a hematopoietic oncoprotein. Blood 107, 1124–1132 16234363 10.1182/blood-2005-03-1196 PMC1895909

[b3-0070265] Eguchi-Ishimae M. Eguchi M. Maki K. Porcher C. Shimizu R. Yamamoto M. Mitani K. (2009). Leukemia-related transcription factor TEL/ETV6 expands erythroid precursors and stimulates hemoglobin synthesis. Cancer Sci. 100, 689–697 19302286 10.1111/j.1349-7006.2009.01097.x PMC11158721

[b4-0070265] Gerety S. S. Wilkinson D. G. (2011). Morpholino artifacts provide pitfalls and reveal a novel role for pro-apoptotic genes in hindbrain boundary development. Dev. Biol. 350, 279–289 21145318 10.1016/j.ydbio.2010.11.030 PMC3111810

[b5-0070265] Gu X. Shin B.-H. Akbarali Y. Weiss A. Boltax J. Oettgen P. Libermann T. A. (2001). Tel-2 is a novel transcriptional repressor related to the Ets factor Tel/ETV-6. J. Biol. Chem. 276, 9421–9436 11108721 10.1074/jbc.M010070200

[b6-0070265] Kawagoe H. Potter M. Ellis J. Grosveld G. C. (2004). TEL2, an ETS factor expressed in human leukemia, regulates monocytic differentiation of U937 Cells and blocks the inhibitory effect of TEL1 on ras-induced cellular transformation. Cancer Res. 64, 6091–6100 15342392 10.1158/0008-5472.CAN-04-0839

[b7-0070265] Mejia-Pous C. Damiola F. Gandrillon O. (2011). Cholesterol synthesis-related enzyme oxidosqualene cyclase is required to maintain self-renewal in primary erythroid progenitors. Cell Prolif. 44, 441–452 21951287 10.1111/j.1365-2184.2011.00771.x PMC6495882

[b8-0070265] Mohandas N. Gallagher P. G. (2008). Red cell membrane: past, present, and future. Blood 112, 3939–3948 18988878 10.1182/blood-2008-07-161166 PMC2582001

[b9-0070265] Paffett-Lugassy N. N. Zon L. I. (2004). Analysis of hematopoietic development in the zebrafish. In Developmental Hematopoiesis: Methods and Protocols (ed. Baron M. H.), pp. 171–198 Totowa, NJ: Humana Press 10.1385/1-59259-826-9:171 15492396

[b10-0070265] Poirel H. Lopez R. G. Lacronique V. Della Valle V. Mauchauffé M. Berger R. Ghysdael J. Bernard O. A. (2000). Characterization of a novel ETS gene, TELB, encoding a protein structurally and functionally related to TEL. Oncogene 19, 4802–4806 11032031 10.1038/sj.onc.1203830

[b11-0070265] Potter M. D. Buijs A. Kreider B. van Rompaey L. Grosveld G. C. (2000). Identification and characterization of a new human ETS-family transcription factor, TEL2, that is expressed in hematopoietic tissues and can associate with TEL1/ETV6. Blood 95, 3341–3348 10828014

[b12-0070265] Quintana A. M. Grosveld G. C. (2011). Zebrafish as a model to characterize TEL2 function during development and cancer. J. Carcinogene Mutagene S1, 1

[b13-0070265] Slupsky C. M. Gentile L. N. Donaldson L. W. Mackereth C. D. Seidel J. J. Graves B. J. McIntosh L. P. (1998). Structure of the Ets-1 pointed domain and mitogen-activated protein kinase phosphorylation site. Proc. Natl. Acad. Sci. USA 95, 12129–12134 9770451 10.1073/pnas.95.21.12129 PMC22796

[b14-0070265] Thisse C. Thisse B. (2008). High-resolution in situ hybridization to whole-mount zebrafish embryos. Nat. Protoc. 3, 59–69 18193022 10.1038/nprot.2007.514

[b15-0070265] Wang L. C. Swat W. Fujiwara Y. Davidson L. Visvader J. Kuo F. Alt F. W. Gilliland D. G. Golub T. R. Orkin S. H. (1998). The TEL/ETV6 gene is required specifically for hematopoiesis in the bone marrow. Genes Dev. 12, 2392–2402 9694803 10.1101/gad.12.15.2392 PMC317042

[b16-0070265] Zapata A. (1979). Ultrastructural study of the teleost fish kidney. Dev. Comp. Immunol. 3, 55–65 437237 10.1016/s0145-305x(79)80006-3

